# Validation of a biomarker tool capable of measuring the absorbed dose soon after exposure to ionizing radiation

**DOI:** 10.1038/s41598-021-87173-3

**Published:** 2021-04-14

**Authors:** Anna Giovanetti, Raffaella Marconi, Noha Awad, Hala Abuzied, Neveen Agamy, Mohamed Barakat, Cecilia Bartoleschi, Gianluca Bossi, Marco Canfora, Amr A. Elsaid, Laura Ioannilli, Horeya M. Ismail, Yasmine Amr Issa, Flavia Novelli, Maria Chiara Pardini, Claudio Pioli, Paola Pinnarò, Giuseppe Sanguineti, Mohamed M. Tahoun, Riccardo Turchi, Lidia Strigari

**Affiliations:** 1 Division of Health Protection Technologies, ENEA-Italian National Agency for New Technologies, Energy and Sustainable Economic Development, 00123 Rome, Italy; 2 Scientific Direction, National Institute for Infectious Diseases “Lazzaro Spallanzani” IRCCS, 00149 Rome, Italy; 3 Epidemiology Department, High Institute of Public Health, Alexandria University, Alexandria, 21561 Egypt; 4 Alexandria University Cancer Research Cluster, Alexandria, 21561 Egypt; 5 Nutrition Department, High Institute of Public Health, Alexandria University, Alexandria, 21561 Egypt; 6 Oncogenomic and Epigenetic Unit, Department of Diagnostic Research and Technological Innovation, IRCCS - Regina Elena National Cancer Institute, 00144 Rome, Italy; 7 Clinical Trial Center, Biostatistics and Bioinformatics, IRCCS Regina Elena National Cancer Institute, 00144 Rome, Italy; 8 Oncology Department, Faculty of Medicine, Alexandria University, Alexandria, 21561 Egypt; 9 Department of Biology, University of Rome “Tor Vergata”, 00133 Rome, Italy; 10 Medical Biochemistry Department, Faculty of Medicine, University of Alexandria, Alexandria, 21561 Egypt; 11 Departments of Radiation Oncology, IRCCS - Regina Elena National Cancer Institute, 00144 Rome, Italy; 12 IRCCS Azienda Ospedaliera Universitaria di Bologna, 40138 Bologna, Italy

**Keywords:** Biomarkers, Prognostic markers, Radiotherapy

## Abstract

A radiological or nuclear attack could involve such a large number of subjects as to overwhelm the emergency facilities in charge. Resources should therefore be focused on those subjects needing immediate medical attention and care. In such a scenario, for the triage management by first responders, it is necessary to count on efficient biological dosimetry tools capable of early detection of the absorbed dose. At present the validated assays for measuring the absorbed dose are dicentric chromosomes and micronuclei counts, which require more than 2–3 days to obtain results. To overcome this limitation the NATO SPS Programme funded an Italian–Egyptian collaborative project aimed at validating a fast, accurate and feasible tool for assessing the absorbed dose early after radiation exposure. Biomarkers as complete blood cell counts, DNA breaks and radio-inducible proteins were investigated on blood samples collected before and 3 h after the first fraction of radiotherapy in patients treated in specific target areas with doses/fraction of about: 2, 3.5 or > 5 Gy and compared with the reference micronuclei count. Based on univariate and multivariate multiple linear regression correlation, our results identify five early biomarkers potentially useful for detecting the extent of the absorbed dose 3 h after the exposure.

## Introduction

A radiological or nuclear (R/N) emergency may occur suddenly and cause severe consequences for the health of the people present and for the environment ^1^ thus, various initiatives have been undertaken to increase the preparedness. Among these, the European network RENEB established a network of experienced laboratories capable of being activated immediately after a radiological emergency. These laboratories will reconstruct the individual absorbed dose using both biological samples and personal electronic devices ^2^. As reported also in RENEB, the validated and most widely used methods for retrospectively measuring the absorbed dose are the dicentric and the micronucleus (MN) count in peripheral lymphocytes ^3,4^. The problem is that the evaluation of the results is possible only after 50 h for the dicentric and 74 h for the MN, being therefore not usable for an appropriate management of the R/N emergencies’ first phase. In case of exposure of a number of people being so high as to overwhelm the medical facilities in charge it is, in fact, necessary to measure the absorbed dose early after irradiation in order to focus resources on the subjects who need immediate medical treatments. The early assessment of the absorbed dose may also aid in predicting the severity of later health outcomes and to put in place early and effective medical countermeasures and treatments.

Studies on earlier biomarkers are limited and recent multiparametric studies in radio-treated patients, based on emerging biomarkers were measured in samples taken from 24 h after the radiation exposure ^5^. HemoDose ^6^, the AFRRI (Armed Forces Radiobiology Research Institute) Biodosimetry Assessment Tool (BAT)^7,8^ and the First-responders Radiological Assessment Triage (WinFRAT) ^9^, are softwares developed for estimating the individuals’ absorbed doses in case of accidental exposure to radiations. All are based both on multi-type blood cell counts and prodromic clinical symptomatology, to be integrated with cytogenetic counts^7,8^. None of these tools appears to provide information on the absorbed dose in the first hours after exposure. Hemodose was also tested ^6^ analysing the WBC counts measured in subjects exposed in historical accidents, finding that a correlation with the dose starts from 24 h and lasts 4 weeks. The granulopoiesis and thrombopoiesis models were in fact found to be more reliable to characterize the cellular dynamics in the late phase (> 15 days), while results in the early phase (< 15 days) are not predictive to estimate the severity of exposure. So, despite the existence of evidence coming from accidental exposure studies, ex vivo radiation models, or animal models ^10,11^, an appropriate human model for reconstructing the absorbed dose few hours after the exposure has not yet been validated.

Variation in complete blood cell count (CBC) has been deeply investigated to the scope and quantitative relationships between the absorbed doses and the absolute lymphocyte counts or lymphocyte depletion rates which have been assessed to be adopted in the R/N emergencies’ management ^6,12–16^. The hematopoietic system, with its high proliferative index is indeed one of the most vulnerable parts of the human body to radiation damage ^17,18^. Although the hematopoietic system’s perturbation occurs immediately after exposure to ionizing radiation (IR) ^6,19^, very few data are available to predict the irradiation extent a few hours after exposure in animal models ^11^. Since, in the event of an accident, it is not possible to make a comparison with the individual’s own baseline level, the results are compared with the population’s average values and the post-irradiation individual. The HemoDose dosimetry tool, was recently validated with the dicentric count by Abend and collaborators ^20^, confirming that is applicable only starting from 24 h after the IR exposure.

There are other biological parameters that have been shown to change soon after the exposure to ionizing radiation. A very early event caused directly by radiation and by the reactive oxygen species (ROS) originating during radiation exposure are DNA breaks ^21^. Because inflammatory mediators ^22^ and cytokines ^23^ are modulated immediately after radiation exposure, they can be included among early biomarkers ^23^. In addition, IR has been also demonstrated to stimulate changes in the gene expression or post-translational modifications of radio-sensitive proteins such as amylase (AML) indicator of irradiation to the parotid gland, Fms-related tyrosine kinase 3 ligand (FLT3-L), a soluble trans-membrane tyrosine kinase which is an important regulator of haematopoiesis is suggested as a biomarker for bone marrow damage and the amino acid citrulline used as index for radio-induced damage to the small bowel ^24–26^.

IR can also trigger an imbalance of trace elements in the body due to the change of valence of the metal ions caused by the high amount of ROS generated very early after the exposure ^27^. Microelements are very abundant in biological systems, and a dose-dependent decrease in serum zinc ^28^ and copper ^29^ have been observed in mice irradiated with increasing doses of gamma rays. These putative early biomarkers have never been evaluated all together, in humans and only 3 h after IR exposure ^30^.

With the aim to set up a novel, fast, accurate and easy to use tool, based on multiple blood parameters approach, for assessing the absorbed dose early after irradiation to a partial-body area, an Italian-Egyptian collaborative study, funded by NATO SPS programme (project: G4815) was carried out. The panel of early biomarkers, including CBC, DNA breaks by Comet assay, AML, FLT3-L, inflammation cytokines as IL1B, IL6, IL 8, zinc and copper concentrations, were validated on cancer patients undergoing radiotherapy (RT) before and 3 h after the first fraction of RT treatment. The obtained results were correlated with the delivered dose to a partial-body area and compared with the results obtained with the reference biomarker MN count assay. In this paper we present the results obtained and their potential use as a predictive tool to early assess the absorbed radiation dose to a partial-body area.

## Results

### Patients’ characteristics

A total of 147 cancer patients naïve for RT have been enrolled by both of the involved Institute/University Hospital and were included in this study. The patients were aged 26 to 97 years (median: 62 years), 84 women and 63 men. The smoke frequency was 27% (i.e. 40/147). The number of patients per dose group that had been calculated initially (see the “Materials and methods” section) was modified following an interim analysis. This analysis was conducted, during the trial, to adjust the sample size, considering the registered early change of biomarkers and the prescribed doses. So, the number of patients per group were modified as followed including 90, 48 and 9 patients respectively for the 2–2.5, 3–3.5 and $\ge$ 5 Gy dose groups.

The patients’ characteristics are reported in Table 1S (see Supplementary Data).

The patient’s irradiated sites were abdomen (41), head and neck (40), thorax (54) and other sites (12). The tumour type and number of patients is detailed in Table 2S (see Supplementary Data).

### Diametric data

**Figure 1:**
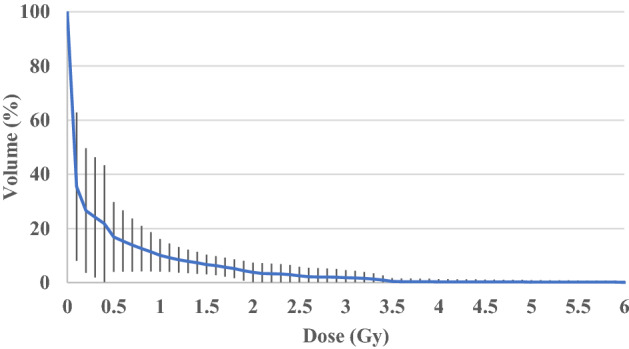
The average planned cumulative body dose volume histograms (solid line) for the whole population. Error bars represent standard deviation of the percentage volume of body receiving a dose equal or greater than or equal to that dose bin (a given range of dose e.g. 0–0.1 Gy).

Details regarding the total number of patients enrolled per the dose group and dose/fraction (expressed in Gy) prescribed at the planning target volume (PTV) are reported in Table 3S (see Supplementary Data). Delivered doses and treated volumes, as well as several parameters extracted from the planned treatment are reported in Table 4S (see Supplementary Data) for the whole populations. Table 4S highlights the high variability of both prescribed and delivered doses per fraction, typically observed in radiotherapy departments. Similarly, a strong variability of the treated volumes is observed both in terms of cm^3^ and in terms of diameter (expressed in cm) of the equivalent sphere (eSPHERE) of PTV or integral dose (expressed in g*Gy): indicates the total energy absorbed by the body, the product of the mass of tissue irradiated and the absorbed dose (Fig. 1).

### Biomarkers results

To get an overview on which early biomarkers show variation between samples taken before (T0) and 3 h after RT (T3), we pooled all the results obtained for all the radiation doses (Table 1). We note however that even in the presence of a high standard deviation (not shown), the increase in the reference biomarker (MN count), that doubled at T3 compared to T0, is very similar to what was observed for DNA breaks (measured with the Comet assay) and with IL8 levels (Table 1). The results obtained at the Rome laboratories at both T0 and T3 did not differ significantly from those obtained at the University of Alexandria in Egypt (data not shown), and were therefore pooled together.

**Table 1:** Median and range values all the biomarkers included in the study are reported measured pre (T0) and post (T3) the first RT fraction.

Biomarker	Median	Minimum	Maximum
WBC—T0	7.04	2.00	19.40
WBC—T3	7.32	3.74	21.54
RBC—T0	4.59	3.16	6.70
RBC—T3	4.54	2.92	6.55
PLT—T0	237	107	548
PLT—T3	236	94	507
NTL—T0	4.26	0.48	15.33
NTL—T3	4.50	1.81	19.39
LYMP—T0	1.94	0.69	9.37
LYMP—T3	2.03	0.79	8.70
MONOC—T0	0.52	0.17	1.57
MONOC—T3	0.48	0.10	1.29
ESN—T0	0.15	0.00	3.38
ESN—T3	0.14	0.00	1.66
BAS—T0	0.04	0.00	0.16
BAS—T3	0.03	0.00	0.14
AML—T0	66.5	17.0	191.0
AML—T3	67.0	15.0	264.0
IL-8—T0	7.1	0.4	144.5
IL-8—T3	7.5	0.0	779.9
IL1b—T0	0.74	0.00	16.50
IL1b—T3	0.78	0.00	30.80
IL-6—T0	1.07	0.00	21.71
IL-6—T3	1.17	0.00	38.89
FLT3—T0	28.4	1.6	285.1
FLT3—T3	31.6	4.1	282.3
Copper—T0	144.3	19.6	323.9
Copper—T3	157.1	61.7	344.3
Comet—T0	44.8	3.5	237.8
Comet—T3	108.0	3.3	379.0
Zinc—T0	1.27	0.15	3.06
Zinc—T3	1.22	0.13	2.44
MN—T0	3.00	0.50	18.00
MN—T3	5.90	1.40	22.50

*WBC* white blood cells, *RBC* red blood cells, *PTL* platelets, *NTL* neutrophils, *LYMP* lymphocytes, *MONOC* monocytes, *ESN* eosinophils, *BAS* basophils, *AML* alpha-amylase, *IL* interleukin, *FLT3* fms related tyrosine kinase 3, *MN* micronuclei.

The absolute increase of DNA breaks (determined by Comet assay) was statistically significantly correlated with the increasing of MN count (p < 0.0001). Correlation with the absolute increase of MN was found for the nominal prescribed dose (p-value = 0.035) but not for the integral dose (p = n.s.). The relative change of IL-6 and DNA breaks was statistically significantly correlated (p-value = 0.036 and 0.0016, respectively) with the relative increase of MN count.

**Figure 2:**
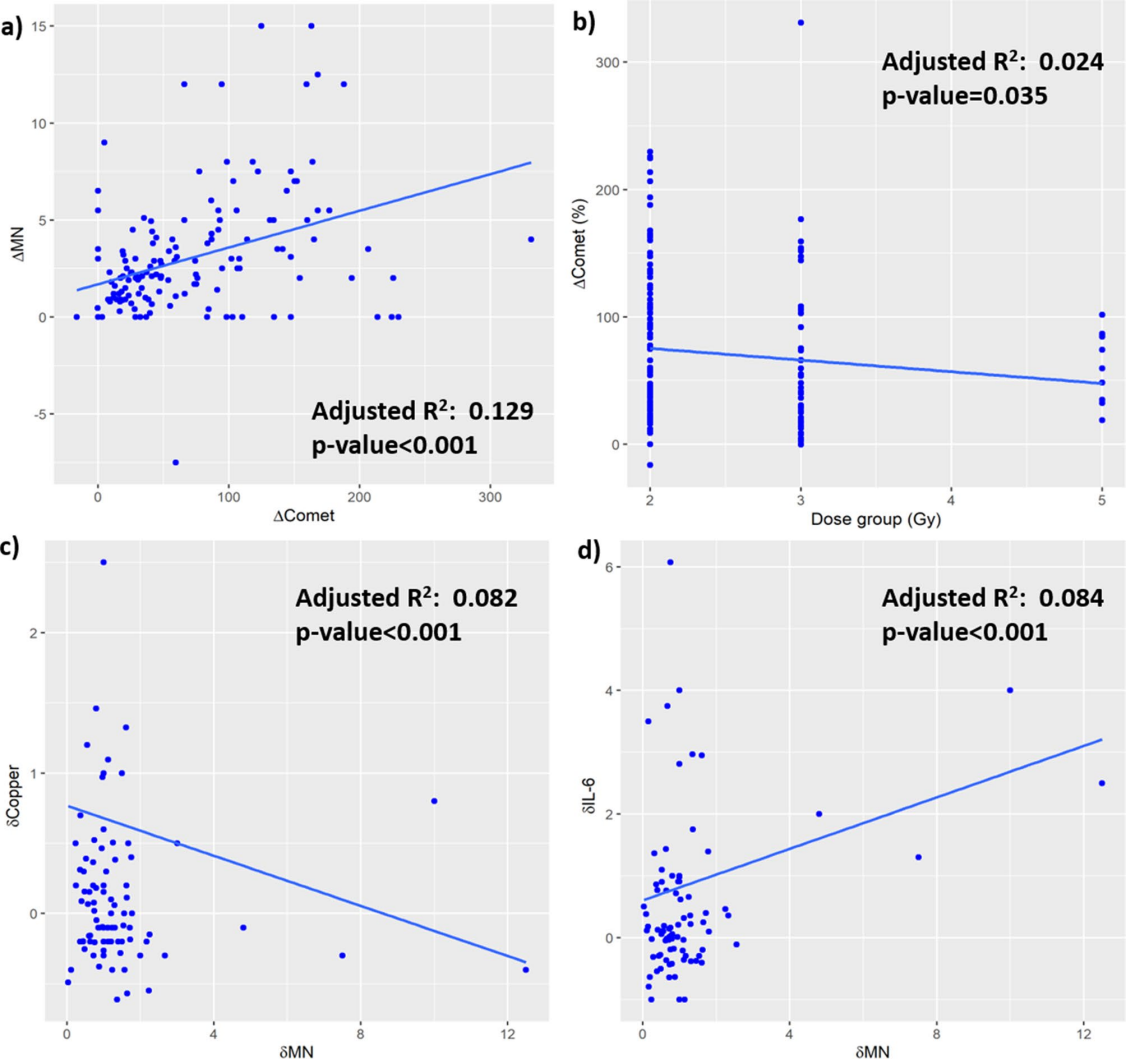
The difference of absolute MN values (before and after the first fraction of radiotherapy) versus the difference of absolute DNA breaks values obtained from Comet assays (**a**) and the PTV dose group (**b**); the change of Copper (**c**) and IL-6 (**d**) values against the percentage values of MN (∆ = absolute difference; δ = relative difference).

**Figure 3:**
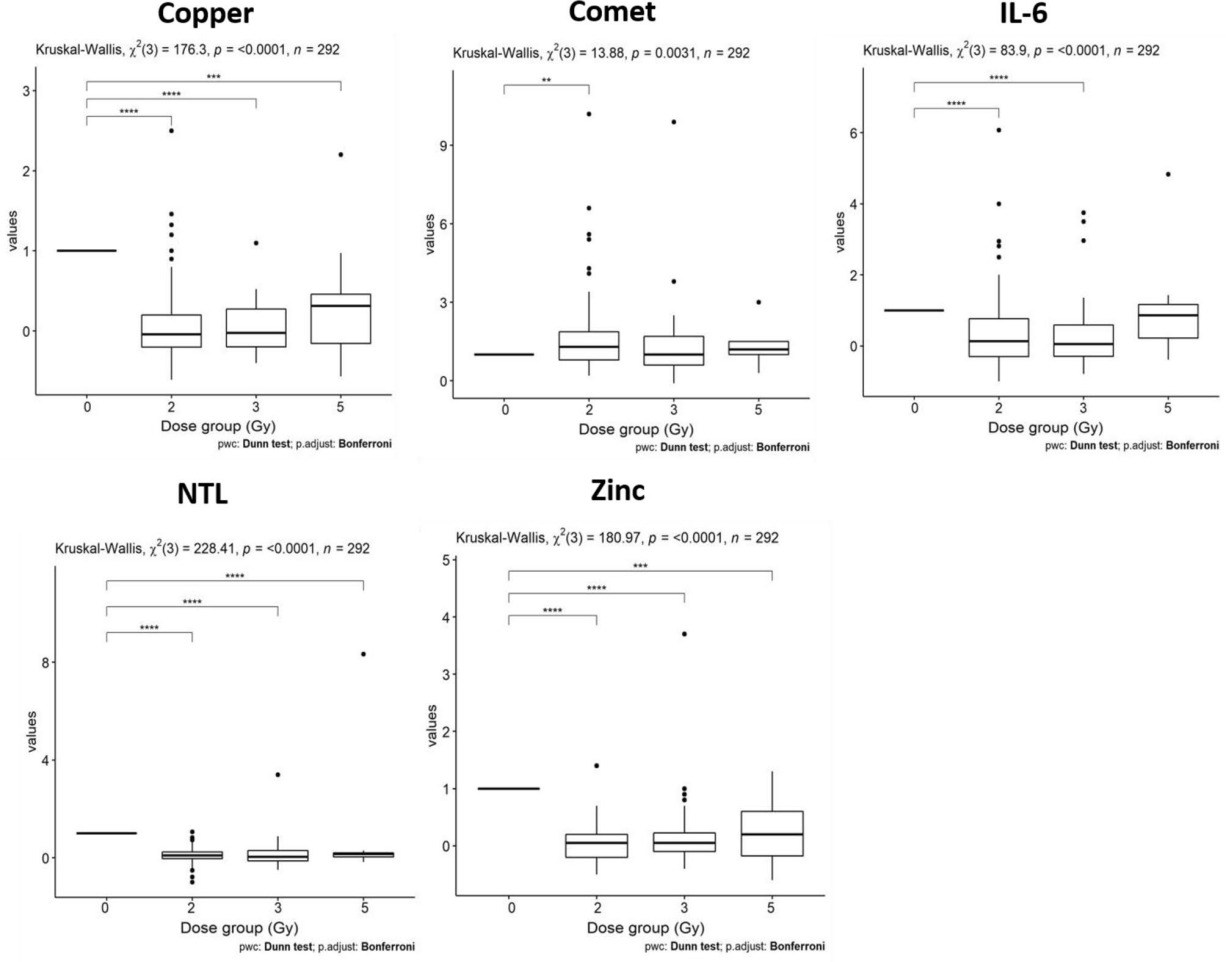
The boxplots of calculated relative change of values of the investigated biomarkers based on the standard test (CBC) according to the PTV dose group (in Gy) representing the dose delivered to a partial-body area. The baseline relative values are indicated as 100%. Boxplots have lines extending from the boxes (whiskers) indicating the 25th and 75th quartiles. Circle dots represent potential outliers.

Based on this preliminary observation, we investigated the relative variation of each biomarker according to the planned tumour volume (PTV) dose group instead of that to the administered integral dose. The behaviour of the values of investigated biomarkers according to the dose group is reported in Figs. 2 and 3 using a logarithmic scale for the vertical axis.

The boxplots of calculated values of the investigated biomarkers based on standard test (CBC) statistically significantly associated to the PTV dose group (in Gy) (i.e. the dose delivered to a partial-body area at the multivariate analysis) are shown in Fig. 3. All the other biomarkers are presented into the supplementary data (Fig. 1S). Comparison Test revealed a significant change in treatment groups compared to control one in most biomarkers.

In addition, the boxplots of Fig. 3 allow us to appreciate in a few biomarkers (e.g., Copper, Zinc) a monotonous trend of the median relative change of values according to the dose group, while for other biomarkers the behaviour seems to be more complicated.

The correlation between early biomarkers and MN encouraged us to construct a novel predictive model for PTV delivered doses to a partial-body area. The partial dose that was delivered to the patients might be representative of the dose received during R/N emergencies not that high as to induce acute effects and not that low as to be considered negligible.

**Figure 4:**
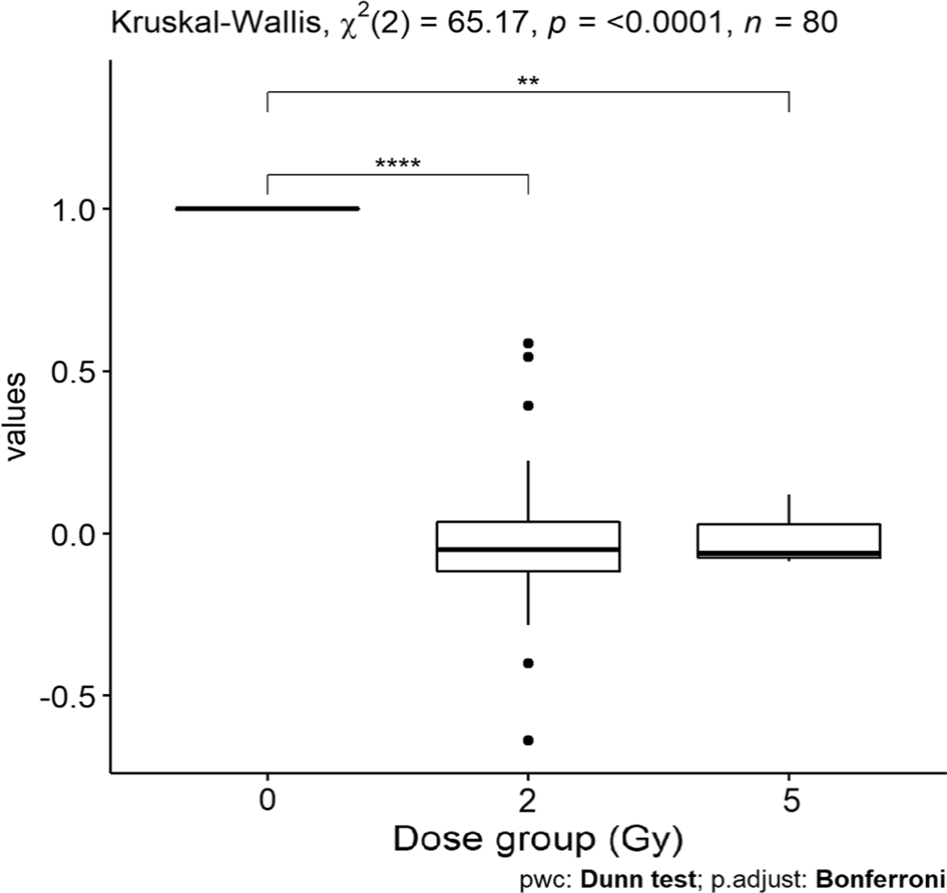
The boxplot of relative change of AML against the dose group in 19 patients for which one or both parotid glands were delineated as organs at risk. The baseline relative values are indicated as 100%.

Since the increase in circulating AML is strictly dependent on the volume of salivary glands irradiated and on the delivered dose ^31^, we decided to analyse separately the 19 patients, for which one or both parotid glands were delineated as organs at risk: 17 patients with H/N cancer, one patient with chemodectoma and one patient with skin cancer. The boxplots of Fig. 4 show the behaviour of relative change of AML values (obtained before and at 3 h post irradiation). In our cohort the median (range) dose at the first fraction to parotid glands was 0.62 Gy (0.09–1.33 Gy). The AML values at T3 are significantly lower comparing the T0 values.

Univariate and multivariate multiple linear regression analysis aimed at predicting the dose group is reported in Tables 2 and 3 for all the calculated values of investigated biomarkers obtained before and 3 h after RT. To construct a possible model for predicting the highest absorbed dose in a part of the body, the correlation between the biomarkers identified as statistically significant at the univariate analysis, was calculated. Only low correlated biomarkers (Parsons correlation coefficient < 0.6) were included as input variable of multivariate multiple linear regression analysis (see Table 3).

**Table 2:** Univariate linear regression analysis of relative change of the selected biomarkers for predicting the dose group.

Independent variables	Coefficient	Standard error	t-value	Adjusted R2	p-value
dWBC	− 195.81	9.89623	− 19.786	0.581	< 0.0001
dRBC	− 235.6	6.98657	− 33.722	0.812	< 0.0001
dPLT	− 224.828	7.1943	− 31.251	0.782	< 0.0001
dLYMP	− 216.218	8.63815	− 25.031	0.694	< 0.0001
dNTL	− 90.3099	10.28292	− 8.783	0.207	< 0.0001
dMONOC	− 160.578	4.92751	− 32.588	0.788	< 0.0001
dESN	− 93.368	8.53853	− 10.935	0.296	< 0.0001
dBAS	− 135.957	4.01015	− 33.903	0.837	< 0.0001
dAML	− 207.685	8.20948	− 25.298	0.706	< 0.0001
dIL6	− 41.5547	10.5484	− 3.939	0.041	0.0001
dIL1b	− 217.142	9.70476	− 22.375	0.695	< 0.0001
dIL8	− 178.847	8.56862	− 20.872	0.723	< 0.0001
dFLT3	1.37323	2.22462	0.617	0.003	0.5378
dCopper	− 163.134	10.38822	− 15.704	0.476	< 0.0001
dComet	25.9189	7.42056	3.493	0.040	0.0006
dZinc	− 170.163	12.16754	− 13.985	0.465	< 0.0001

**Table 3:** Multivariate multiple linear regression analysis of the relative variation of the selected biomarkers for predicting the dose group in Gy.

Independent variables	Multivariate linear regression analysis			
	Coefficient	standard error	t-value	p-value
dNTL	68.43389	4.97579	13.753	< 0.0001
dIL6	− 38.1885	5.44159	− 7.018	< 0.0001
dCopper	6.07267	2.10292	2.888	0.0045
dComet	− 87.5857	13.34696	− 6.562	< 0.0001
dZinc	− 156.11	9.42986	− 16.555	< 0.0001

At the univariate linear regression analysis, the calculated values of most of the analysed biomarkers (e.g. dWBC, dRBC, dPLT, dLYMP, dNTL, dMONOC, dESN, dBAS, etc.) decrease when the dose per fraction rises (Table 3), inversely the dCOMET increases.

The multivariate multiple linear regression analysis produced the following function to predict delivered doses to a partial-body area:

$${\text{Predicted \; dose }}\left( {{\text{cGy}}} \right) \, = 207.8 + 68.43389 \times {\text{dNTL}} - 38.1885 \times {\text{dIL6}} + 6.07267 \times {\text{dCopper}} - 87.5857 \times {\text{dComet}} - 156.11 \times {\text{dZinc}};$$

with a R-adjusted of 0.9681, a multiple correlation coefficient of 0.9845 and a F-ratio of 949 (p < 0.001).

To evaluate the validity of our prediction the Area under the Receiver Operating Characteristic (ROC) has been calculated: If Area Under Curve (AUC) = 1, it means there is perfect prediction by the model; If AUC = 0.5, it would mean the model is unable to discriminate between dose classes. The Area under the ROC curve of predicted delivered doses to a partial-body area was 0.962 with a 95% confidence interval from 0.930 to 0.982 (p < 0.0001).

## Discussion

In the event of an R/N emergency it is necessary to know as soon as possible whether the subjects present have been exposed to a dose level that requires medical treatment. Since the cytogenetic biomarkers or the softwares used for the retrospective calculation of the dose are not utilizable before 24 h, we wanted to test, on patients undergoing RT, the possible use of early biomarkers. Five biomarkers, independently of each other, have been demonstrated to be significantly correlated with the absorbed dose: NTL, DNA breaks (by Comet assay) and the concentration of IL6, Copper and Zinc.

The selected early biomarkers have been validated using as reference the MN count, widely applied for the retrospective calculation of the absorbed dose ^3^. We detected an increase of about 75% of MN compared to the baseline value at each dose per fraction. The inter-individual variability, including that before RT (data not shown), was high, due to the fact that the participants are cancer patients. Confounding factors may be represented by genetic polymorphism, previous radiological pre-therapeutic diagnostic evaluation (e.g. PET, CT) or other environmental factors.

The delivered doses were 2–2.5, 3–3.5 and $\ge$ 5 Gy. In our model, the predicted dose is related to the maximal dose delivered to PTV variable from 2.6 to 1728 cm^3^ representative of partial-body irradiation, corresponding to an integral dose from 0.0098 to 0.5769 g*Gy. Since the cardiac output is equal to a blood circulation speed of 5 l/min ^32^, considering a total blood volume of 7 l (average man at rest) we assumed that blood recirculates in about 60 s in the target volume thus suggesting that biomarkers might be independent on the irradiated volume. Considering that the irradiation time varies from 2 to 5 min, the whole blood volume is irradiated multiple times during the RT delivery. In this context, the PTV delivered doses may be treated as a partial-body exposure as occurs during the R/N emergencies ^33^ requiring the triage, being intermediated between the higher doses inducing acute radiation syndrome and dose levels not requiring immediate medical interventions.

Radio-induced variation in WBC counts has been deeply investigated and empirical formulas ^14,34^ have established a quantitative relationship with the absorbed doses. As previously reported, the estimation of the absorbed dose using the software based on the WBC count as HemoDose, is possible only starting from 24 h ^6^ In this study applying the univariate linear regression analysis, we found a significant correlation between the decrease of WBC, RBC, PLT, LYMP, BAS and ESN with the increasing of the dose per fraction only 3 h after RT.

At multivariate regression analysis only five biomarkers were found to show a significant independent correlation with the delivered target dose, namely neutrophils ratio, IL-6, DNA breaks (by Comet assay) and metal ions (copper and zinc) status being consistent with the variation of MN count.

The dose-dependent neutrophils depletion observed 3 h post RT, may be due to the lack of DNA repair proteins in this type of cells, primed to undergo spontaneous cell death ^35^. In fact, the neutrophils’ lifespan in circulating blood, in fact, ranges from hours to a few days ^36^.

DNA breaks were demonstrated to be a sensitive marker of IR exposure ^37^ and increase in workers chronically exposed to low-doses of IR ^38^. Our results confirm the significant correlation between the amount of DNA breaks, directly caused by IR or by radio-induced reactive oxygen species (ROS), and the radiation absorbed dose. In our cohort we observed an average increase of 0.026 DNA breaks per Gy. Most DNA breaks we assessed with Comet assay are single strand breaks (SSB) that easily repair, differently from the double strand breaks (DSB) that if not repaired or miss-repaired cause the formation of chromosomal aberrations, which may lead to human diseases including cancer ^21^. Because the early formation DNA breaks, the rapidity of the protocol and the very reduced amount of blood required, the Comet assay is a good early biomarker, but since SSB are soon repaired, this is not a feasible indicator for retrospective studies conducted more than 24 h after radiation exposure. In fact, measuring DSB with the corresponding γ-H2AX foci, a maximum of 35γH2AX/Gy was observed in vitro 3 min post-IR exposure decreasing 30 min after at 20γH2AX/Gy ^39^. After 1 h the number of γH2AX foci was found to decrease rapidly to about 50% ^40^. Thus, the correlation between Gy and DNA breaks we found after 3 h is likely lower than what we would have found within 1 h, but even so, it is correlated with the absorbed dose and can be used as a feasible early biomarker.

Inflammation mediators are reported as being up- or down-regulated during radiation responses and release of ILs such as IL-6 and IL-8 was observed for different types of tumours following exposure to gamma-rays ^22^. IR indeed was demonstrated to stimulate the inflammatory response through the transient activation of key transcription factors as nuclear factor kappa B (NF-kB). NF-kB in turn, plays a key role in inflammation and immune responses by regulating the expression of pro-inflammatory cytokines and chemokines. Cytokine production usually reaches its peak at 4–24 h after irradiation, then decreases to the baseline levels from 24 h ^22^. Accordingly, we observed a very early increase of interleukins in our in vivo study likely related to the irradiation of normal tissue surrounding the target with a heterogeneous dose distribution.

Among the proteins, the concentration of which has been indicated to vary due to the gene expression or post-translational modifications induced by IR, we analysed the variation in concentration of AML, an indicator of radiation damage to the parotid gland ^31^, and FLT3L, used in estimating the severity of the haematopoietic syndrome in radiation accident victims ^24^. In 65 patients and in non-human primates before and after fractionated RT the plasma concentrations of AML, FLT3L and MCP1 (monocyte chemotactic protein) were found to be significantly higher, Balog et al. ^41^, starting from 24 h after the last irradiation. Amylase activity was demonstrated to rise, in head-and-neck (H/N) cancer patients, at 9–36 or 24–48 h after irradiation ^31^. In our RT practice, to reduce the mean dose of parotid glands (and fulfil the dose constraint of 26 Gy) a dose gradient around the PTV was generated in particular where the PTV encloses part of the parotid glands, while in the studies reported in literature (e.g. using total body irradiation) the dose to the parotid gland was homogeneously delivered (up to 12 Gy). Our results cannot be, therefore, directly compared. Three hours after RT no evidence of AML modulation was found. However, when AML plasma level was analysed only in the patients in which one or both the salivary glands have been irradiated, a significant decrease was found 3 h after RT comparing to the values at T0. This result is apparently in contrast to what is reported by other authors and is not easily explained. One hypothesis might be that we measured the AML earlier.

For FLT3L, even if it has been classified as an early biomarker, the contribution to the dose prediction was showed to start from 48 h post exposure ^26^. Thus, the lack of modulation that we have observed possibly derived from the fact that 3 h from irradiation is too short a time.

In agreement with what has been reported by Min et al. ^28^ in mice, we found that the serum Zn^2+^ level decreases considerably by increasing the delivered dose. This can be attributed to the change of the valence state of zinc from Zn^2+^ to Zn^+^ caused by the radio-induced free radicals ^27^. A dose-related decreasing was also found for Cu^2+^, confirming the results obtained in irradiated mice ^29^. Serum iron increases with increasing dose and serum copper is strongly associated with the change in serum iron. Serum Cu^2+^ indeed combines mainly with ceruloplasmin, which oxidizes the ferrous ions into ferric ions. This oxidation process changes the serum copper to a monovalent state ^29^, as shown on mice for zinc ^28^. The change of metal valence status appears to be a suitable biomarker for IR exposure because the dose-related decreasing starts immediately after irradiation and remains with the same values for at least 21 days ^28^.

The realization of this study was possible only by involving patients who had to undergo radiotherapy. This implied limitations due to the great variability of basal values induced by the disease and other treatments and the exclusion of some radiation biomarkers as C reactive protein, the free circulating DNA, proven to rise in cancer patients. Noteworthy is the fact that the evaluation of multiple biomarkers, based on different biological mechanisms allows the reducing of the potential bias. To reduce the inter-patient variability, the relative biomarkers’ variation has been calculated and correlated with the delivered dose. Despite these limitations, we found with multivariate regression analysis five biomarkers to show a significant correlation with the delivered target dose. Another point of strength of our model is that it is based on an accurate patient dosimetry obtained using the treatment planning system and dose verification in clinical treatments. However, further data sets are needed to validate these five biomarkers for their application in the early management of R/N emergencies. By applying the calculation of the results used to minimize the baseline individual variability and using the same formula for the results obtained in Egypt, the data were comparable and it was possible to analyse them together.

## Conclusions

The main result of our study is, in the both Institutions involved, the correlation of several early biomarkers with the absorbed dose according to the RT treatment plans. We recognize that these candidate biomarkers must be independently validated on further data sets. Nevertheless, the identified early biomarkers might represent an important step towards the development of a tool capable of establishing the dose level shortly after a possible R/N emergency. As with all biomarkers used after accidental exposure, the problem is the lack of individual baseline values. This limitation would not exist in the case of occupational or military exposures, where the biomarkers that we have identified to vary with the dose, could be included in the periodically scheduled blood tests. They should then be measured soon after possible radiation exposure.

## Material and methods

### Ethics statement

Cancer patients undergoing RT have been enrolled in Italian and Egyptian Institutions in a dedicated clinical study approved by the corresponding Institutional Ethics Committee at the Istituti Fisioterapici Ospitalieri—Istituto Nazionale Tumori “Regina Elena” (IRE-IFO) (RS No 619/14) and at Alexandria University (approved 13/05/2015), in agreement with the Helsinki declaration. A written informed consent was obtained from all subjects before blood sampling. Blood tubes were provided anonymised, and their identification was only accessible to the project’s principal investigator.

### Patient enrolment criteria and irradiation

Patients were recruited at the Radiotherapy Department of IRE-IFO, Rome (Italy) and at Alexandria Hospital, Alexandria (Egypt). Eligibility criteria for patients’ enrolment were: over 18 years of age; capable and willing to give informed consent; not previous RT; scheduled for a first dose of either 2 Gy, 3 Gy or ≥ 5 Gy; not currently or recently (≥ 1 year) undergoing chemotherapy; without haematological malignancy or other blood or metabolic diseases. Data related to biomarkers cancer features as proposed in the study and previous therapy and follow up (if any) have been retrieved from medical records and recorded in a dedicated web-based database (in compliance with sensitive data protection requirements) specifically developed for this study.

### Data protection

Data regarding experimental, dosimetric and clinical information were anonymized and collected in a dedicated web-based database accessible only to authorized staff.

### Trial sample size

The number of patients to enrol was calculated assuming a difference of about 20% in the reference biomarker (MN count) obtained before and after the first session of treatment (in other words, we assumed a basal value of 20% and an increase up to 40% after irradiation);

- assuming an alpha value of 0.05 and a power of 80% the minimum number is 36;
- assuming an alpha value of 0.01 and a power of 80% the minimum number is 52.

Summarizing in each Institute/University the enrolment was: 50 patients exposed to a single dose of 2 Gy of radiations; 15 patients exposed to a single dose of about 3 Gy of radiations; 5 patients exposed to a single dose ≥ 5 Gy.

### Patient blood collection and plasma preparation

Peripheral blood was collected before and at 3 h after RT by peripheral puncture using BD Vacutainer (BD LIFE SCIENCES, Franklin Lakes, NJ, US). For blood count, Comet assay, MN count and plasma vacutainers were used with lithium heparin as the anticoagulant (N cat 367884); vacutainers with a clot activator (N cat 367986) were used for serum analysis. When not indicated otherwise the reagents were purchased from SIGMA ALDRICH (St. Louis, MO, USA).

### Cell blood counts

Fresh whole blood samples were collected in EDTA, immediately and CBC obtained with Beckman coulter DXH800. Our reference methodology and equipment have been provided by the Clinical Pathology Laboratory at IFO, that is UNI EN ISO 9001:2015 (IT276208 re 24/07/2018 valid until 05/09/2022) certified for all diagnostic activities performed and for the planning of research activities in oncology. In addition, IFO is also authorized for Phase I Clinical Study by the Italian Medicines Agency (AIFA) as well as OECI certificated. All diagnostic analyses are performed in compliance with the principles of Good Laboratory Practice (GLP) to ensure the generation of high quality and reliable test data. For CBC counts the Egyptian partners involved 13 different laboratories, to standardize these measurement aliquots of blood from the same healthy subjects were sent to all of these laboratories.

### α-Amylase test

Tests were performed at the Clinical Pathology Laboratory at IFO. 5 ml of peripheral blood were collected in EDTA and centrifuged 10 min at 2000×*g*, within 2 h after sampling, then serum was aliquoted and frozen at − 80 °C until analyses. Samples were then processed by colorimetric enzymatic in vitro assay AMYL2, ACN 570—STAT, reaction time: 7, (ROCHE DIAGNOSTICS, Basel, CH) and p-Nitrophenol (4-Nitrophenol), generated by a-Amylase dissociation and a-glucosidase hydrolysis, quantified (mkat/l) with COBAS 8000 System (ROCHE DIAGNOSTICS, Basel, CH), majoring increased absorbance (at 700/415 nm), which is directly proportional to the a-Amylase activity.

### Reference MN count assay

1.5 ml of whole blood were split into three T25 flasks and grown in 1640 RPMI with 15% FBS and 1% pen/strep; after 44 h Cytochalasin B was added (final concentration of 6 μg/ml) and 72 h from the seeding, cells were fixed. Briefly after centrifuge 1000×*g* × G10 min) cells were rinsed with PBS, centrifuged and incubated for 2 min at 37 °C with 0.075 M KCl hypotonic solution, then centrifuged and fixed with cold methanol/acetic acid 5:1; this step was replicated twice using methanol/acetic acid 3:1. After staining with 10 ug/ml ethidium bromide, MN were scored at × 400 magnification by a fluorescent Axiolab Zeiss microscope (CARL ZEISS AG, Oberkochen, Germany) and the % of MN on 500 binucleated cells was counted.

### DNA breaks by Comet assay

The alkaline Comet assay was performed as described by Giovanetti et al. ^42^. Briefly, 20 μl of whole blood were embedded in an agarose and lysed at 4 °C ((2.5 M NaCl, 10 mM Tris–HCl, 100 mM Na^2^EDTA, NaOH to pH 10, 1% Triton, 10%DMSO) for 45 min. Then after rinsing with electrophoresis buffer (1 mM Na^2^EDTA, 300 mM NaOH, pH 13) slides were placed onto the electrophoresis unit Subcell GT System/15 × 25 cm equipped with Power Pack 300 (BIO RAD LABORATORIES INC, Hercules, CA, USA) containing the same buffer for 20 min and electrophoresed for 30 min (26 V, 300 mA). Lastly, the slides were neutralised, dehydrated with ethanol series and after staining with EtBr (10 μg/ml) the Comets were analysed at × 400 magnification by a fluorescent Axiolab Zeiss microscope (CARL ZEISS AG, Oberkochen, Germany). The amount of DNA breaks was assessed by Visual scoring method ^43^.

*Inflammatory cytokines* were assessed on patients’ sera using the BDTM Cytometric Bead Array (CBA, kit BD551811, BECTON DICKINSON, Franklin Lakes, NJ, USA), a kit allowing quantitative analysis of soluble analytes by flow cytometry. Procedures were performed according to the manufacturer’s instructions. Briefly, the CBA assay contained six bead populations with distinct fluorescence intensities that were distinguished in the FL3 channel (LP670 filter) of the FACSCalibur flow cytometer (BECTON DICKINSON, Franklin Lakes, NJ, USA). Each bead population recognized a determined cytokine through a capture antibody specific for either IL-8, or IL-1β, or IL-6, or IL-10, or TNF, or IL-12p70. Captured cytokines were bound by phycoerythrin (PE)-conjugated anti-cytokine specific antibodies to form sandwich complexes. PE-fluorescence detected on FL2 channel (PB585/42 filter) was proportional to the cytokine concentration in the sample. Recombinant cytokines provided with the kit were used to obtain a reference standard curve with known serially-diluted cytokine concentrations (5000–2.5 pg/ml). According to preliminary experiments, serum samples were diluted 1:2. Both FSC/SSC and fluorescence parameters were acquired in log mode. No less than 3000 events/sample were acquired by Flow cytometer (> 500 events/cytokine/sample). Data were analysed with FCAP Array software (SOFT FLOW Kft, Pecs, H). Samples collected from patients in Alessandria (Egypt) were also analysed at the ENEA laboratories.

*FLT3-L concentration* was measured with the ab100521—FLT3 Ligand Human ELISA Kit (ABCAM, Cambridge, UK). Following the producers’ Instruction without modifications. This assay employs an antibody specific for Human FLT3-L coated on a 96-well plate. 100 μl of each standard, diluted as suggested by producers, and 100 μl of undiluted serum samples were pipetted into wells, that were then washed and biotinylated with anti-Human FLT3-L antibody. After washing away the unbound biotinylated antibody, HRP-conjugated streptavidin was pipetted into the wells. The wells are again washed and a TMB substrate solution is added to the wells, the colour develops in proportion to the amount of bound FLT3-L. The Stop Solution changes the colour from blue to yellow, and the intensity of the colour is measured at 450 nm with spectrophotometer (DAS s.r.l., Palombara Sabina, IT). The samples’ absorbance values were related by linear regression to the Standard curve ones.

*Zinc concentration in serum* was determined with the Zinc Microplate assay kit (cat N. MBS8243228) (MYBIOSOURCE San Diego, CA, US) following the producer’s instructions. The standard curve was obtained by 8 serial 1:2 dilutions of the 100 μM/l standard solution provided by the producer. The absorbance was read out with spectrometer (DAS s.r.l., Palombara Sabina, IT) plate reader at 550 nm. All samples and standards were tested in duplicate.

*Copper concentration in serum* was determined using the Copper Microplate Assay kit (cat N. MBS8292800) (MYBIOSOURCE San Diego, CA, US) following the producer’s instructions. The colorimetric readout at 605 nm wavelength was measured by GloMax Discover Microplate Reader (PROMEGA, Madison, WI, US). Standard and samples were measured against blank with 500 µMol/l of standard concentration. A standard curve was run for each assay.

### Patient dosimetry

The baseline patients’ characteristics and dosimetric information (as eSphere, planning tumour volume, PTV, body volume, etc.) have been described as median (range) or frequency table as appropriated. PTV i.e. the planning target volume (expressed in cubic cm) identifies the volume treated at higher doses during the radiotherapy treatment. The eSphere indicates the diameter (in cm) of a sphere having the same volume of a region of interest in this case the PTV. Integral dose (expresses in gram × Gray): indicates the total energy absorbed by the body in the non-target tissues, the product of the mass of tissue irradiated and the absorbed dose.

More in details, based on DICOM data of each single patient, the integral dose Ij to the body j divided into n voxels is given by:

$$I_{j} = \sum\limits_{i}^{n} {D_{ij} m_{ij} = \sum\limits_{i}^{n} {D_{ij} \upsilon_{ij} \rho_{ij} } ,}$$

where D_ij_, m_ij_, v_ij_ and ρ_ij_ are the dose, mass, volume and density of voxel i in organ j, according to the formalism reported by D’Souza and Rosen ^44^. Plans were generated and delivered using Eclipse treatment planning system v. 13.5 and Clinac 2100/CD (VARIAN MEDICAL SYSTEMS, Palo Alto, CA, US) at the IRCCS Regina Elena, Rome, respectively, and using the Peacock treatment planning system (NOMOS Corp., Sewickley, PA, US) and the Elekta Synergy linac (ELEKTA AB, Stockholm, Sweden) at the Medical Research Institute, Alexandria University, respectively. The dose volume histograms (ss) and dose distribution were extracted from each treatment planning system (TPS) and imported in the software VODCA version 5.4. to homogenize the methodology of DVHs calculation of the integral dose. Thus, the DVHs were converted in “comma-separated values (csv)” format. The DVHs is a histogram relating the tissue volume receiving a given dose of radiotherapy. A cumulative DVHs is created by first determining the size of the dose bins of the histogram while the column height of the first bin (0–0.1 Gy) represents the percentage volume of structure receiving a dose equal or greater than or equal to that dose bin (e.g. 0–0.1 Gy). For a structure receiving a very homogenous dose (100% of the volume receiving exactly 6 Gy, for example) the cumulative DVH will appear as a horizontal line at the top of the graph, at 100% volume as plotted vertically, with a vertical drop at 6 Gy on the horizontal axis.

Data Analysis/Statistic methodology. For each patient and biomarker, three measurements were obtained. All the biomarkers evaluated within this study in blood samples obtained before irradiation were used as the baseline control values (n = 147). Moreover, to take into consideration the biomarker variability of each patient, the percentage variation was determined as the difference between values after the first fraction of RT minus baseline, divided by the baseline value (expressed in percentage). The mean and the standard deviation of biomarkers and volumes were determined. The Pearson correlation test ^45^ was used to test a possible relationship between radiation dose and biomarkers’ modification.

Statistical analysis of more than three groups (i.e. the control and the dose groups) was performed using one-way analysis of variance followed with the Bonferroni test for parametric data and Kruskal–Wallis test followed with Dunnett's multiple comparison test for non-parametric data.

Univariate linear regression analysis and multivariate multiple linear regression analysis was conducted to find factors relevant to identify absorbed dose. To fulfil the assumption of linear regression model, data were log-transformed. To assess the goodness of regression model e.g. to measure of the precision with which the regression coefficient is measured the t statistic (i.e. the ratio of coefficient divided by its standard error) was calculated. If a coefficient is large compared to its standard error, then the variable has a genuine effect on the dependent variable.

All the variable significant at the univariate linear regression analysis using a cut-off of 0.05 were included in the multivariate multiple linear regression analysis. Since multicollinearity inflates the variance of coefficients and causes type II errors, we removed the highly correlated variables with a correlation coefficient higher or equal to 0.6.

Receiver operating characteristic (ROC) analysis was used to calculate the area under the curve (AUC) of the model developed for identifying the dose group of irradiated subjects. Data analyses were done using the comprehensive statistical analysis package known as R-package (version R3.5.2) and the Matlab Statistics Tool (version R2019a) (MATHWORKS, Natick, MA, US).

## Supplementary Information

Supplementary Information.

## References

[1] Gale RP (2017). Medical and policy considerations for nuclear and radiation accidents, incidents and terrorism. Curr. Opin. Hematol..

[2] Brzozowska B (2017). RENEB accident simulation exercise. Int. J. Radiat. Biol..

[3] Vral A, Fenech M, Thierens H (2011). The micronucleus assay as a biological dosimeter of in vivo ionising radiation exposure. Mutagenesis.

[4] Depuydt J (2017). RENEB intercomparison exercises analyzing micronuclei (cytokinesis-block micronucleus assay). Int. J. Radiat. Biol..

[5] Tichy A (2018). The first in vivo multiparametric comparison of different radiation exposure biomarkers in human blood. PLoS ONE.

[6] Hu S, Blakely WF, Cucinotta FA (2015). HEMODOSE: A biodosimetry tool based on multi-type blood cell counts. Health Phys..

[7] Blakely WF, Madrid JP, Sandgren DJ (2010). Biodosimetry medical recording-use of the biodosimetry assessment tool. Health Phys..

[8] Sandgren DJ (2010). Biodosimetry Assessment Tool (BAT) software-dose prediction algorithms. Health Phys..

[9] First-responders Radiological Assessment Triage (WinFRAT), version 0.7.6.0 beta. (Accessed 17 July 2020); https://www.usuhs.edu/afrri/biodosimetrytools (2013).

[10] Prasanna PG (2010). Synopsis of partial-body radiation diagnostic biomarkers and medical management of radiation injury workshop. Radiat. Res..

[11] Sproull M, Camphausen K (2016). State-of-the-art advances in radiation biodosimetry for mass casualty events involving radiation exposure. Radiat. Res..

[12] Bolduc DL (2019). Baboon radiation quality (mixed-field neutron and gamma, gamma alone) dose-response model systems: Assessment of H-ARS severity using haematologic biomarkers. Radiat. Prot. Dosimetry..

[13] Sine RC (2001). biodosimety assessment tool: A post-exposure software application for management of radiation accidents. Mil. Med..

[14] Goans RE, Waselenko JK (2005). Medical management of radiological casualties. Health Phys..

[15] Smirnova OA (2012). Comparative analysis of the dynamics of thrombocytopoietic, granulocytopoietic, and erythropoietic systems in irradiated humans: A modeling approach. Health Phys..

[16] REMM Radiation emergency medical management. *Dose Estimator for Exposure: 3 Biodosimetry Tools* (Accessed 17 July 2020); www.remm.nlm.gov/ars_wbd.htm (2018).

[17] Goans RE (2001). Early dose assessment in critically accidents. Health Phys..

[18] Fliedner TM, Graessle DH (2007). Hematopoietic cell renewal systems: Mechanisms of coping and failing after chronic exposure to ionizing radiation. Radiat. Environ. Biophys..

[19] Williams EK (1954). The white cell count in relation to occupational radiation dosage. Acta Radiol..

[20] Haupt J, Ostheim P, Port M, Abend M (2020). Using dicentric dose estimates and early radiation-induced blood cell count changes of real case histories for validation of the hemodose biodosimetry tool. Radiat. Prot. Dosimetry.

[21] Vignard J, Mirey G, Salles B (2013). Ionizing-radiation induced DNA double-strand breaks: A direct and indirect lighting up. Radiother. Oncol..

[22] Multhoff G, Radons J (2012). Radiation, inflammation, and immune responses in cancer. Front. Oncol..

[23] Ossetrova NI (2018). Biomarkers for radiation biodosimetry and injury assessment after mixed-field (neutron and gamma) radiation in the mouse total-body irradiation model. Health Phys..

[24] Guipaud O, Benderitter M (2009). Protein biomarkers for radiation exposure: Towards a proteomic approach as a new investigation tool. Ann. Ist. Super. Sanità.

[25] Huang J (2019). Proteomic profiling for serum biomarkers in mice exposed to ionizing radiation. Dose-Response.

[26] Ossetrova NI, Sandgren DJ, Blakely WF (2014). Protein biomarkers for enhancement of radiation dose and injury assessment in nonhuman primate total-body irradiation model. Radiat. Prot. Dosimetry..

[27] Yanin SN (2015). Changing the properties of metals under conditions of exposure to ionizing radiation. IOP Conf. Ser. Mater. Sci. Eng..

[28] Min XY (2014). Development of serum zinc as a biological dosimeter in mice. Int. J. Radiat. Biol..

[29] Zhang XH (2013). Development of serum copper-based biological dosimetry in whole body gamma irradiation of mice. Health Phys..

[30] Giovanetti, A. *et al.* Investigating early biomarkers of radiation exposure to estimate absorbed dose/patient radiosensitivity. In *Proc. 4th International CBRNe Workshop, Countering Radiological and Nuclear Threats, 8 Nov 2018*. ( Accessed 17 July 2020); http://www.aracneeditrice.it/index.php/collana.html?col=CBRNe (2019).

[31] De Felice F (2017). Radiation therapy and serum salivary amylase in head and neck cancer. Oncotarget.

[32] Silverthon DU (2019). Human Physiology: An Integrated Approach.

[33] IAEA. Criteria for use in preparedness and response for a nuclear or radiological emergency. In *Safety Standards Series* No. GSG-2 (2011).

[34] Baranov AE, Guskova AK, Nadejina NM, Nugis VY (1995). Chernobyl experience: Biological indicators of exposure to ionizing radiation. Stem Cells.

[35] Baranov V (2019). Compromised DNA repair and signalling in human granulocytes. J. Innate Immun..

[36] Strydom N, Rankin SM (2013). Regulation of circulating neutrophil numbers under homeostasis and in disease. J. Innate Immun..

[37] Berthel E, Ferlazzo ML, Devic C, Bourguignon M, Foray N (2019). What does the history of research on the repair of DNA double-strand breaks tell us? A comprehensive review of human radiosensitivity. Int. J. Mol Sci..

[38] Møller P, Møller L, Godschalk RW, Jones GD (2010). Assessment and reduction of Comet assay variation in relation to DNA damage: Studies from the European Comet Assay Validation Group. Mutagenesis.

[39] Asaithamby A, Chen DJ (2009). Cellular responses to DNA double-strand breaks after low-dose gamma-irradiation. Nucleic Acids Res..

[40] Rothkamm K, Horn S (2009). gamma-H2AX as protein biomarker for radiation exposure. Ann. Ist. Super. Sanità.

[41] Balog RP (2020). Development of a biodosimeter for radiation triage using novel blood protein biomarker panels in humans and non-human primates. Int. J. Radiat. Biol..

[42] Giovanetti A, Deshpande T, Basso E (2008). Persistence of genetic damage in mice exposed to low dose of X rays. Int. J. Radiat Biol..

[43] Azqueta A (2011). The influence of scoring method on variability in results obtained with the Comet assay. Mutagenesis.

[44] D'Souza WD, Rosen II (2003). Nontumor integral dose variation in conventional radiotherapy treatment planning. Med. Phys..

[45] Pearson K, Henrici OMFE (1896). VII. Mathematical contributions to the theory of evolution. III. Regression, heredity, and panmixia. Philos. Trans. R. Soc. Lond. Ser. A..

