# Supplementary material for: Validation of a biomarker tool capable of measuring the absorbed dose soon after exposure to ionizing radiation
Source: Sci Rep. 2021 Apr 14;11:8118. doi: 10.1038/s41598-021-87173-3 (PMC8047015; doi:10.1038/s41598-021-87173-3)
Supplement: Supplementary file 1 — Supplementary Information. [file 41598_2021_87173_MOESM1_ESM.docx]

Validation of a biomarker tool capable of measuring the absorbed dose soon after exposure to ionizing radiation.

Anna Giovanetti^1^*, Raffaella Marconi^2^, Noha Awad^3^, Hala Abuzied^4^, Neveen Agamy^5^, Mohamed Barakat^4^, Cecilia Bartoleschi^1^, Gianluca Bossi^6^, Marco Canfora^7^, Amr A. Elsaid^8^, Laura Ioannilli^9^, Horeya M. Ismail^4^, Yasmine Amr Issa^10^, Flavia Novelli^1^, Maria Chiara Pardini^1^, Claudio Pioli^1^, Paola Pinnarò^11^, Giuseppe Sanguineti^11^, Mohamed M. Tahoun^3^, Riccardo Turchi^9^, Lidia Strigari^12^

^1^ Division of Health Protection Technologies, ENEA-Italian National Agency for New Technologies, Energy and Sustainable Economic Development, Rome, 00123, Italy.

^2^ Scientific Direction, National Institute for Infectious Diseases "Lazzaro Spallanzani" IRCCS, Rome, 00149, Italy.

^3^ Epidemiology Department, High Institute of Public Health, Alexandria University, Alexandria 21561, Egypt.

^4^ Alexandria University Cancer Research Cluster, Alexandria, 21561, Egypt.

^5^ Nutrition Department, High Institute of Public Health, Alexandria University, Alexandria, 21561, Egypt.

^6^ Oncogenomic and Epigenetic Unit, Department of Diagnostic Research and Technological Innovation, IRCCS - Regina Elena National Cancer Institute, Rome, 00144, Italy.

^7^ Clinical Trial Center, Biostatistics and Bioinformatics, IRCCS Regina Elena National Cancer Institute, Rome, 00144, Italy.

^8^ Oncology Department, Faculty of Medicine, Alexandria University, Alexandria, 21561, Egypt.

^9^ Department of Biology, University of Rome "Tor Vergata", Rome, 00133, Italy.

^10^ Medical Biochemistry Department, Faculty of Medicine, University of Alexandria, Alexandria, 21561, Egypt.

^11^ Departments of Radiation Oncology, IRCCS - Regina Elena National Cancer Institute, Rome, 00144, Italy.

^12^ IRCCS Azienda Ospedaliera Universitaria di Bologna, Bologna, 40138, Italy.

**Supplementary data**

**Table 1S**: Characteristics of patients enrolled in the study at IRE-IFO and Alexandria University Hospital

| **Parameter** | **Median (range)** | **Frequency** |
| --- | --- | --- |
| Age (years) | 62 (26-97) |  |
| Height (cm) | 163 (140-185) |  |
| Weight (kg) | 73 (44-123) |  |
| Sex (N. male/N. female) |  | 63/84 |
| Smoke (yes/no) |  | 40/107 |

**Table 2S**: Tumour types and number of enrolled patients in the study at IRE-IFO and Alexandria University Hospital

| **Tumour type** | **# of enrolled patient(s)** |
| --- | --- |
| Bladder ca | 3 |
| Breast ca | 50 |
| Bone metastasis | 7 |
| Brain tumors ° | 19 |
| H&N cancer* | 21 |
| Lung ca | 3 |
| Other§ | 5 |
| Prostate ca | 20 |
| Rectum ca | 5 |
| Sarcomas | 5 |
| Skin ca | 3 |
| Uterus ca | 6 |

° n.4 glioblastomas, n.2 meningiomas, n.1 pituitary tumor, n.12 brain metastases

* n.6 oral cavity, n.4 larynx, n.8 nasopharynx, n.1 hypopharyngeal cancer

§ n.1 non-Hodgkin's lymphoma, n.1 myeloma, n.2 lymph-node metastases

**Table 3S**: Dose per fraction of Planning Target Volume (PTVs) of investigated RT treatment

| **Dose/fraction (Gy)** | **Dose group (Gy)** | **Number of enrolled patients** |
| --- | --- | --- |
| 2.0-2.5 | 2 | 90 |
| 3.4-4.0 | 3 | 48 |
| ≥ 5.0 | 5 | 9 |
| All doses |  | 147 |

**Table 4S**: Mean doses after one fraction and volumes obtained from DVHs

| **Parameter** | **Median (range)** |
| --- | --- |
| PTV mean dose (Gy) § | 3.4 (1.8-34.51) |
| PTV volume (cm3) | 326.5 (2.6 -1727.7) |
| eSphere (cm) | 8.54 (1.71- 14.89) |
| Body volume in the CT images (cm^3^) | 20442 (536- 52018) |
| Integral dose (g*Gy) | 0.1035 (0.0098-0.5769) |

§ After 1 fraction

Abbreviations: PTV (planning target volume), (expressed in cubic cm), identifies the volume treated at higher doses during the radiotherapy treatment; eSphere indicates the diameter (expressed in cm) of a sphere having the same volume of a region of interest in this case the PTV; Integral dose (expressed in gram * Gy) indicates the total energy absorbed by the body, the product of the mass of tissue irradiated and the absorbed dose.

To further explain the variability observed at the voxel level in our population we reported average DVH (dose volume histogram) of the treated body.

The cumulative DVH variability after one fraction of RT is shown in Fig. 1 reporting the standard deviation of percentage volumes calculated for the whole population at each dose bin. The decreasing percentage of volume receiving higher doses indicates a very heterogeneous dose distribution.


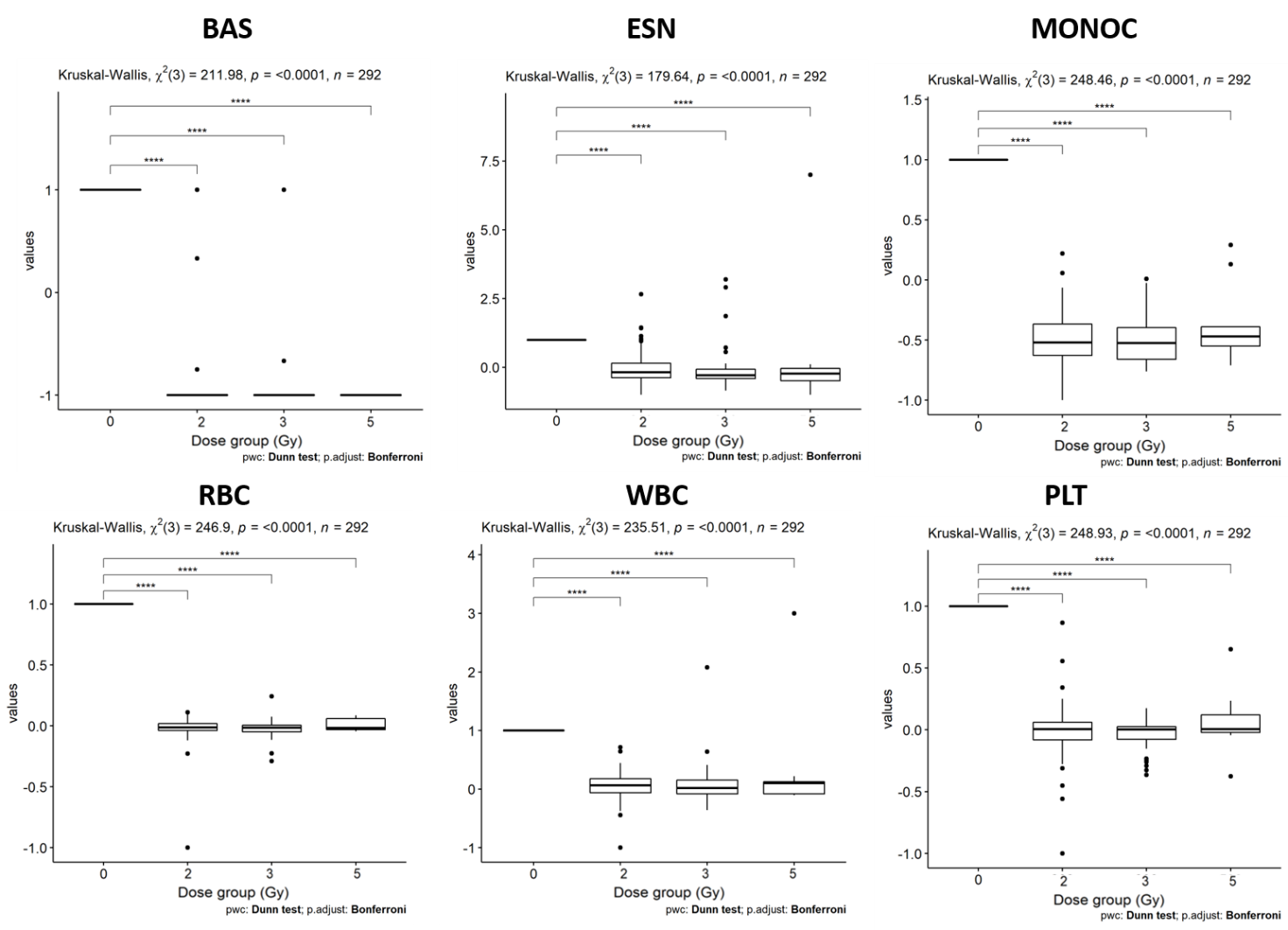


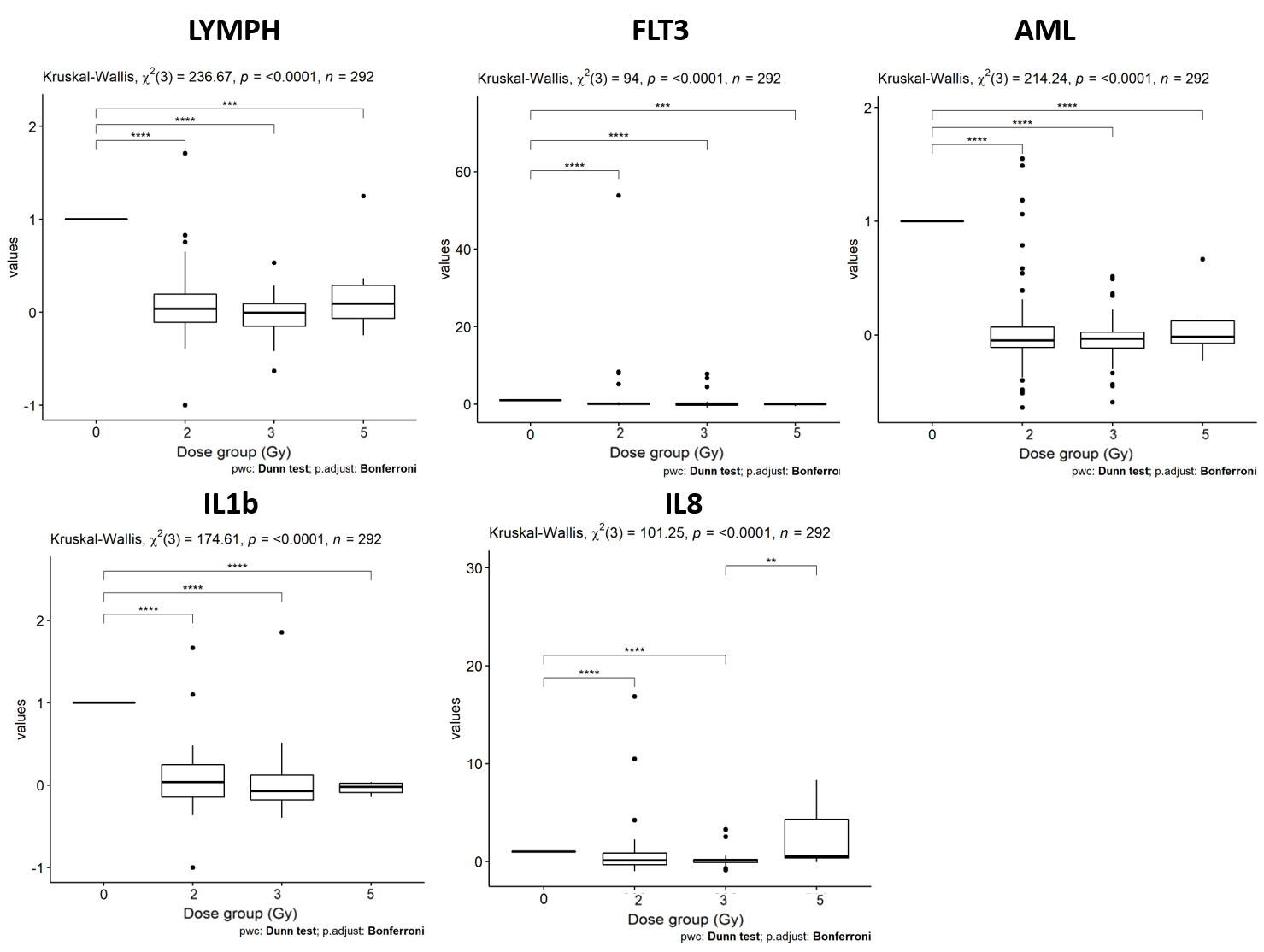


**Fig 1S**: The boxplots of calculated relative change of values of the biomarkers that at the multivariate analysis were not found to be statistically significantly related to the dose group, based on standard test (CBC) according to the PTV dose group (in Gy). The baseline relative values are indicated as 100%. Boxplots have lines extending from the boxes (whiskers) indicating the 25th and 75th quartiles. Circle dots represent potential outliers.
